# Supplementary material for: A sex-specific thermogenic neurocircuit induced by predator smell recruiting cholecystokinin neurons in the dorsomedial hypothalamus
Source: Nat Commun. 2023 Aug 15;14:4937. doi: 10.1038/s41467-023-40484-7 (PMC10427624; doi:10.1038/s41467-023-40484-7)
Supplement: Supplementary file 1 — Supplementary Information [file 41467_2023_40484_MOESM1_ESM.pdf]

SUPPLEMENTARY FIGURES 1-8

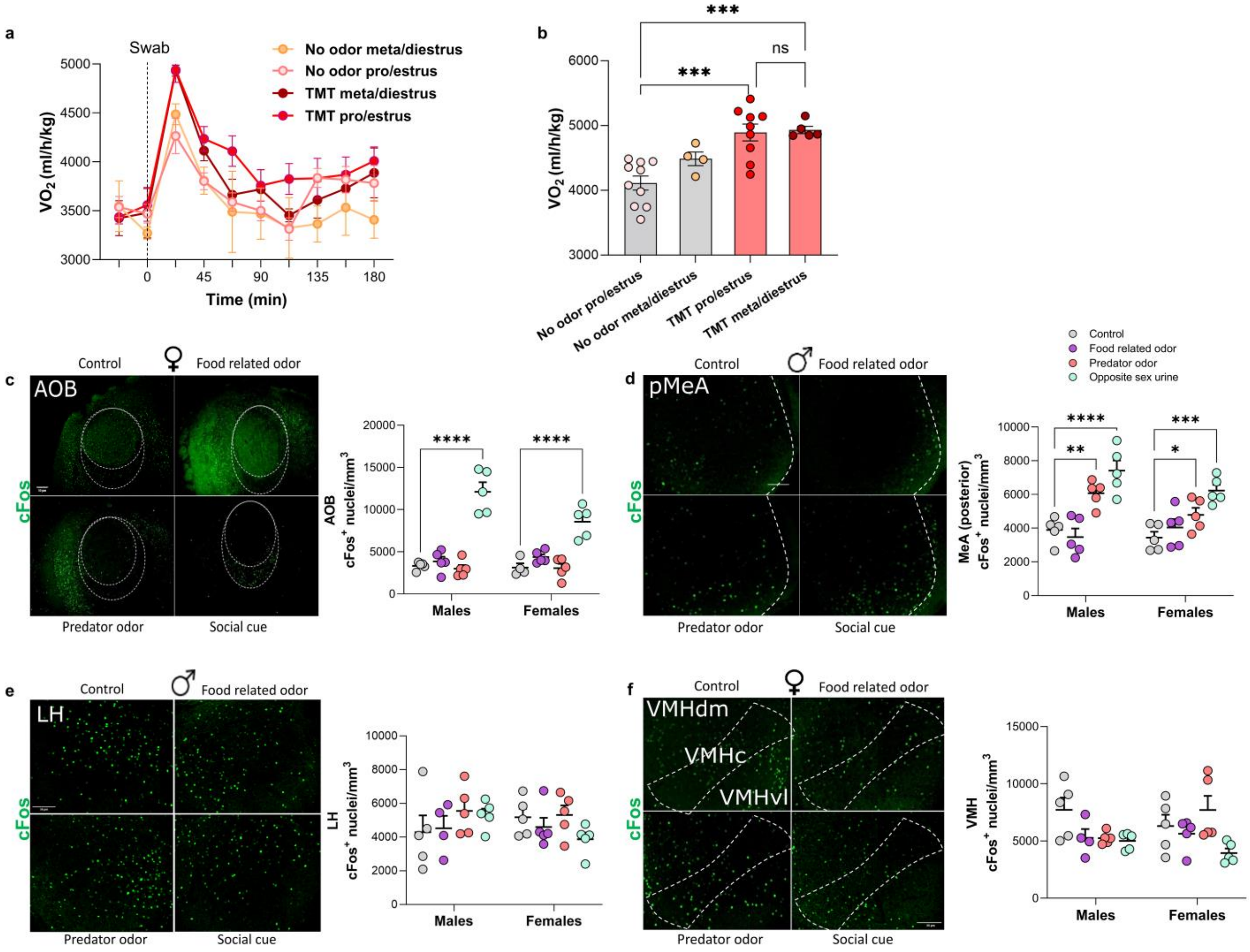

Supplementary figure 1. Assessment of different estrus cycle phase effects on oxygen consumption upon exposure to predator smell and effect of predator smell on neuronal activity in mediobasal hypothalamus. a. Oxygen consumption 3 hours post-exposure to TMT in females divided into groups by their estrus cycle phase, No odor, N=10/pro-estrus and N=4/meta-diestrus, TMT, N=9/pro-estrus and N=5/meta-diestrus. **b.** Oxygen consumption at first time point post-exposure to TMT, No odor, N=10/pro-estrus and N=4/meta-diestrus, TMT, N=9/pro-estrus and N=5/meta-diestrus, Two-way Anova with Sidak's post hoc comparison. **c,d,e, f.** cFos expression after odor exposure in AOB, pMeA, LH and VMH, respectively, N=5 per group, N=4 per group for AOB control females, LH and VMH food odor males, Two-way Anova with Sidak's post hoc comparison, scale 100µm. All bar graphs are presented as mean values ± SEM. Table S8 contains the detailed results of the statistical analysis. *p < 0.05, **p < 0.01, ***p < 0.001, ****p < 0.0001. Source data are provided as a Source Data file.

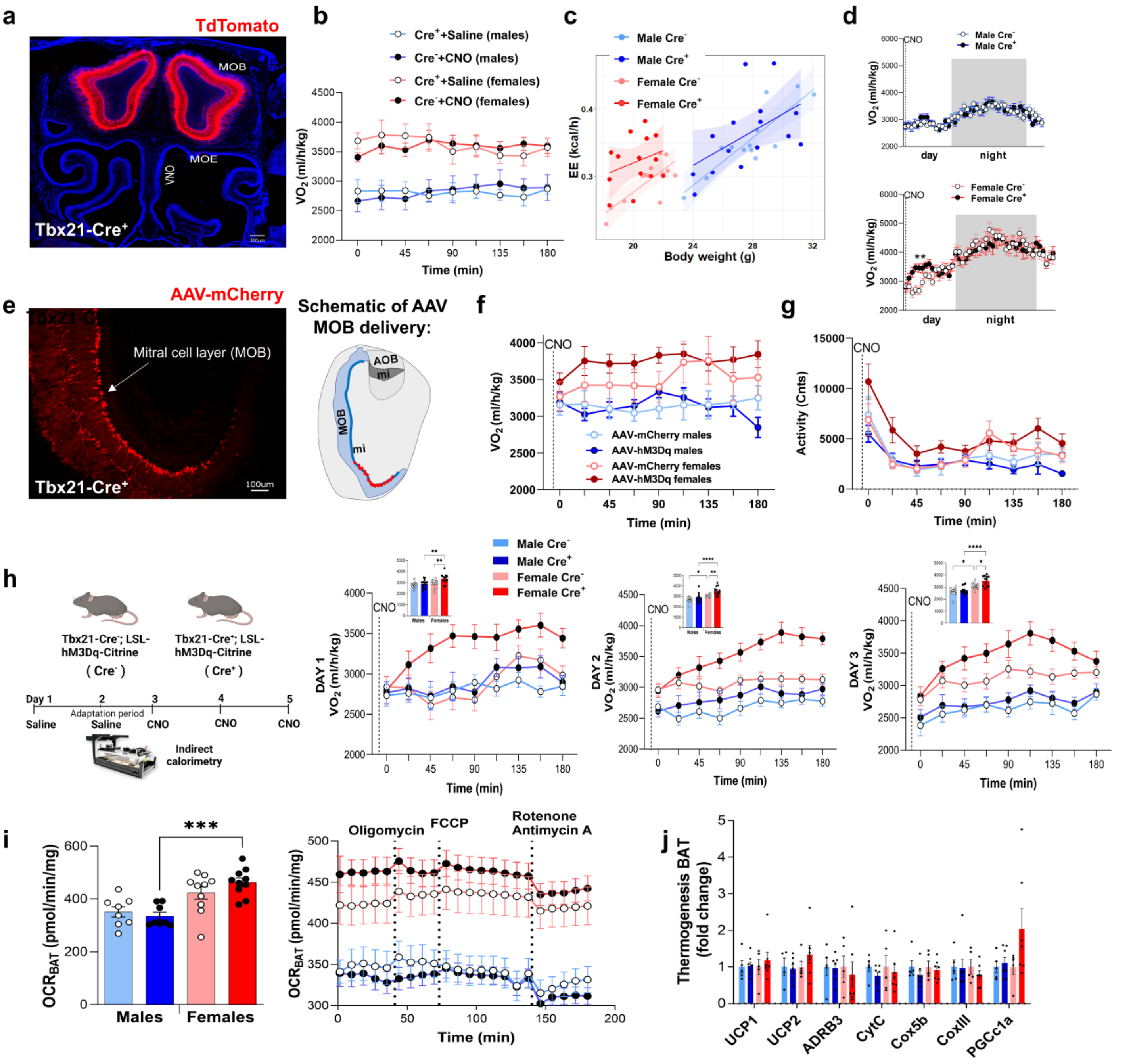

Supplementary figure 2. Sexually dimorphic modulation of energy expenditure upon activation of mitral and tufted olfactory cells. a. Representative picture of MOE and MOB of Tbx21-Cre$^+$/LSL-TdTomato$^+$ mouse, TdTomato (red), scale 300µm. **b.** Oxygen consumption 3 hours post-injection of saline or CNO, males, N=5 per group, females, N=5/Cre$^+$+Saline and N=6/Cre$^-$+CNO. **c.** Analysis of covariance (ANCOVA) of energy expenditure (EE) following CNO injection with body weight as covariance, males, N=14/Cre$^-$ and N=15/Cre$^+$, females, N=13/Cre$^-$ and N=14/Cre$^+$, SD: males, Cre$^-$ 0.047, Cre$^+$ 0.055, females, Cre$^-$ 0.031, Cre$^+$ 0.037. **d.**

Oxygen consumption measured during day and night phases following CNO injection, blue (males), red (females), males, N=14 per group, females, N=13 per group. **e.** Representative picture of mCherry expression in mitral cell layer of MOB upon injection of AAV-DIO-mCherry in MOB of Tbx-Cre mice, mCherry (red) and schematic representation of virus delivery in MOB, Scale bar 100µm. **f,g.** Oxygen consumption and activity 3 hours post-injection of CNO, males, N=6 per group, females, N=5/AAV-mCherry and N=7/AAV-hM3Dq. **h.** Experimental design. Tbx21-Cre^{+}/LSL-$TdTomato^{+}$ or control mice were treated with saline prior to CNO that was administered for three consecutive days, big panel: Oxygen consumption over three days of consecutive injections of CNO, small panel: Average oxygen consumption measured for 3 hours post-injection of CNO, N=14 per group, females, N=13 per group, Two-way Anova with Tukey's post hoc comparison. **i.** Average oxygen consumption rate (left panel) and oxygen consumption rate of brown adipose tissue measured post-injection of CNO (right panel), males, N=8 per group, females, N=10 per group, Two-way Anova with Tukey's post hoc comparison. **j.** RTqPCR against thermogenesis genes in brown adipose tissue of mice post-injection of CNO, males, N=5/Cre^{-} and N=7/Cre^{+}, females, N=5/Cre^{-} and N=7/Cre^{+}. All bar graphs are presented as mean values ± SEM. Table S9 contains the detailed results of the statistical analysis. *$p < 0.05$, **$p < 0.01$, ***$p < 0.001$, ****$p < 0.0001$. Source data are provided as a Source Data file.

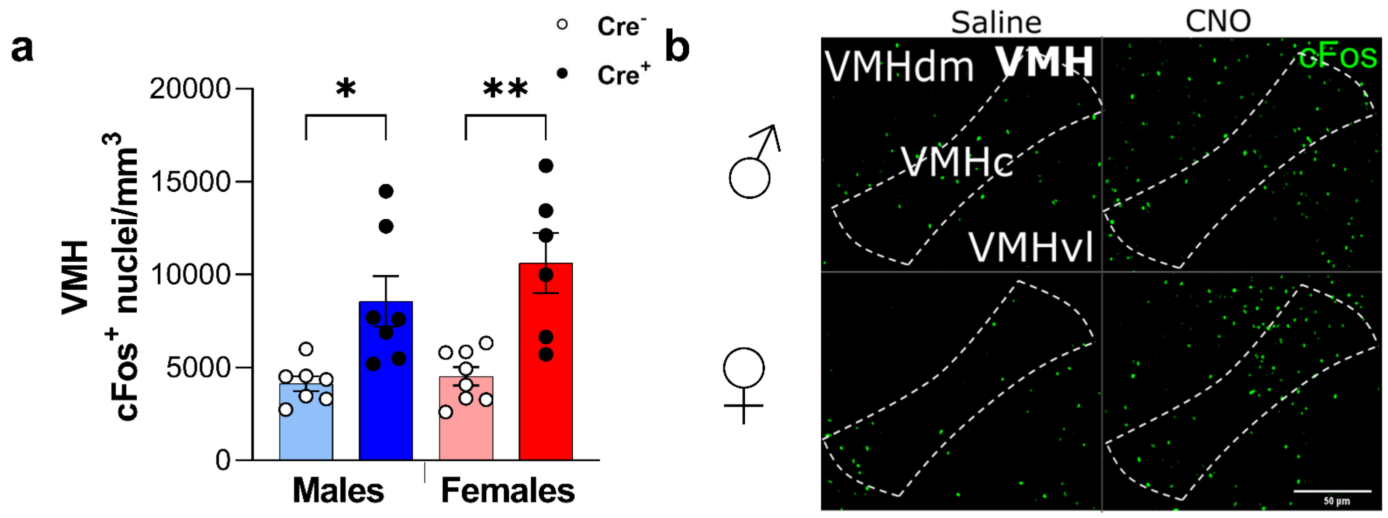

Supplementary figure 3. cFos expression in VMH post-injection of CNO. a. Quantification of cFos expression in VMH following CNO injection, males, N=7 per group, females, N=8/Cre$^-$ and N=6/Cre$^+$, Two-way Anova with Tukey's post hoc comparison. **b.** Immunostaining of cFos (green) in VMH, scale 50µm. All bar graphs are presented as mean values ± SEM. Table S10 contains the detailed results of the statistical analysis. *$p < 0.05$, **$p < 0.01$. Source data are provided as a Source Data file.

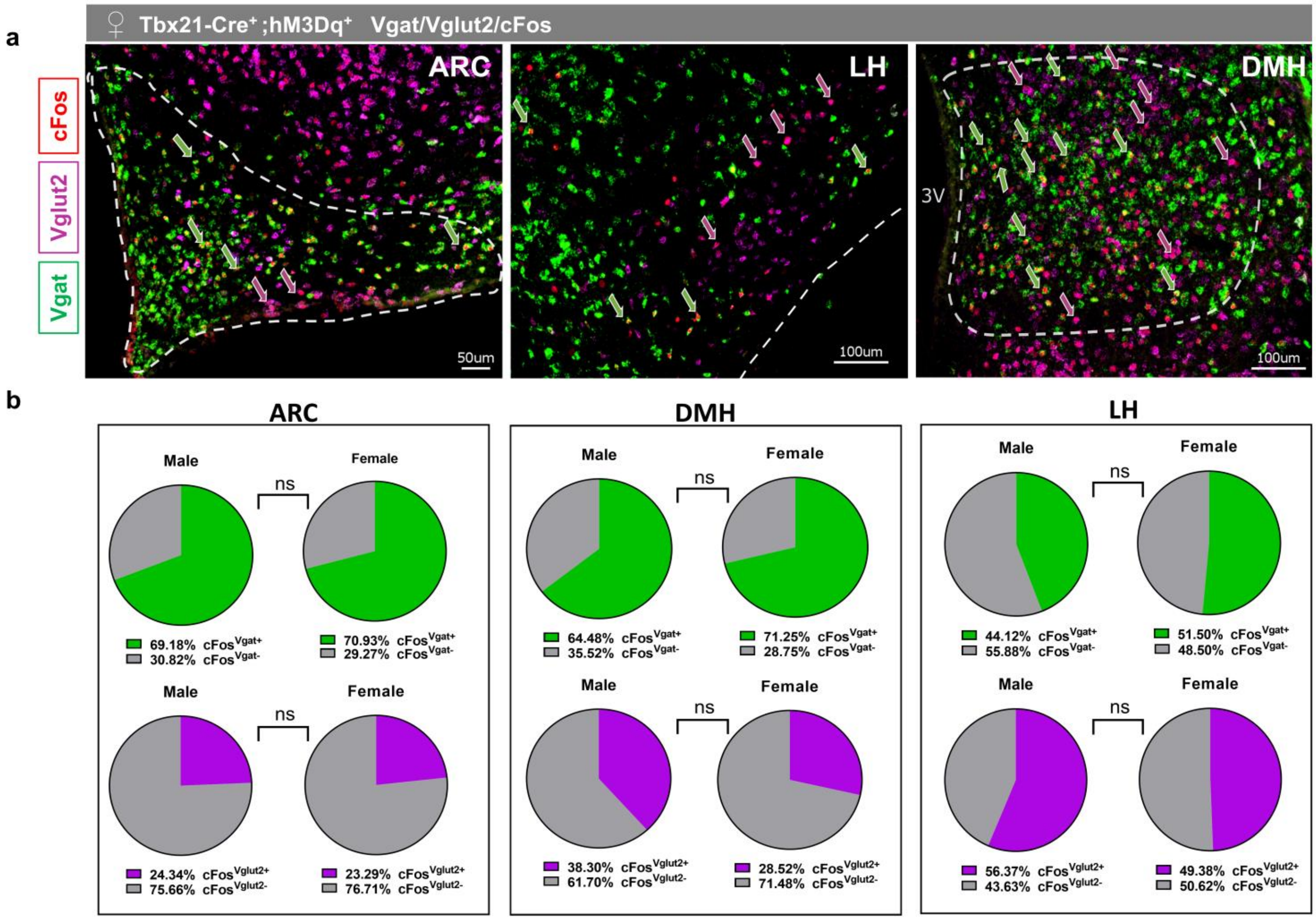

Supplementary figure 4. Recruitment of GABAergic and glutamatergic MBH neurons by chemogenetic stimulation of MOB neurons. a. Representative images of immunohistochemical and in situ hybridization co-staining in female ARC, LH and DMH for Vgat (green), Vglut2 (magenta) and cFos protein (red), green arrows point to colocalization of Vgat/cFos signal, pink arrows point to colocalization of Vglut2/cFos signal, Scale bar 50µm and 100µm. **b.** Quantification of colocalization between Vgat/cFos and Vglut2/cFos signal in ARC, LH and DMH of male and female mice, N=5 per group, N=4/ARC males, Unpaired t-test, Two-tailed. Source data are provided as a Source Data file.

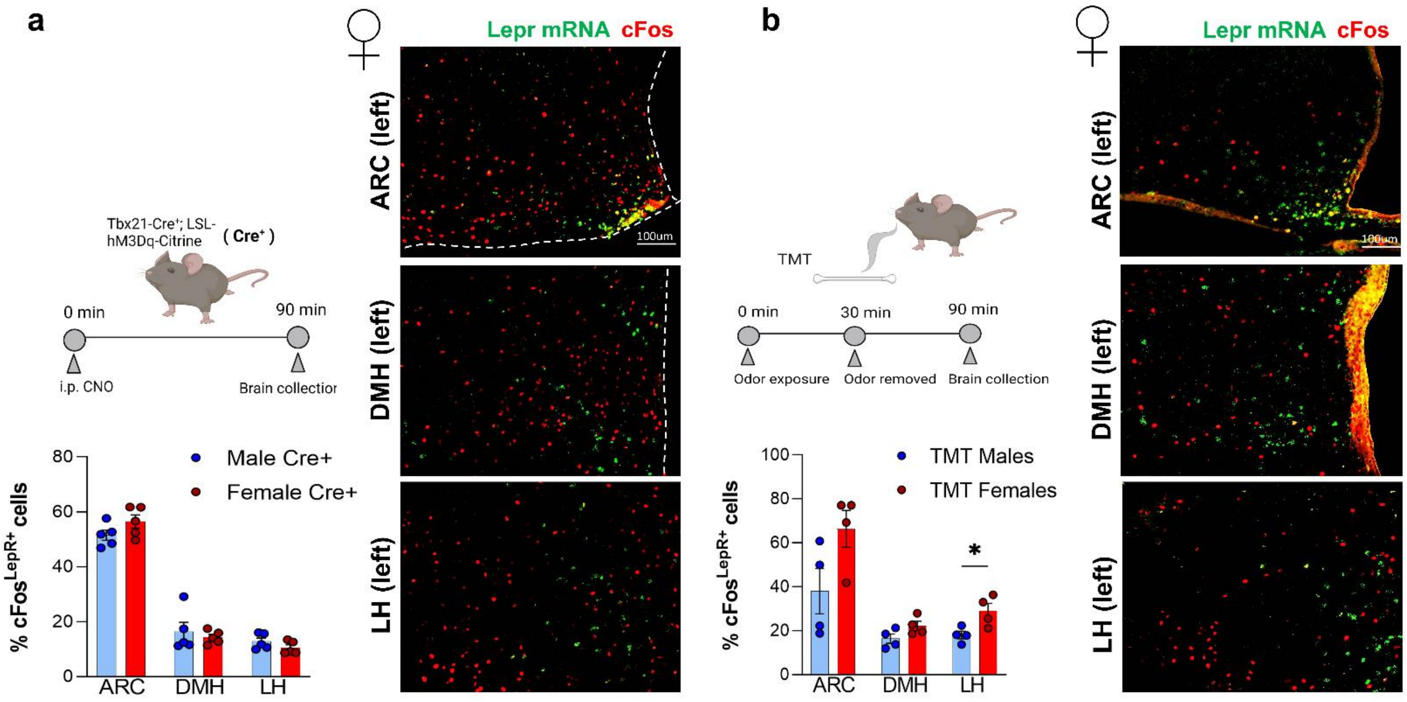

Supplementary figure 5. Activation of LepR-expressing neurons upon olfactory stimuli. a. Left upper panel: Experimental design. Brain tissue of Cre^{+}-hM3Dq^{+} and control mice were collected 90 minutes post-injection of CNO, Left lower panel: Quantification of LepR/cFos signal in ARC, DMH and LH, N=5 per group, Right panel: Representative images of immunohistochemical and in situ hybridization co-staining in female ARC, DMH and LH for LepR (green) and cFos protein (red), yellow cells express LepR/cFos, scale 100µm. **b.** Left upper panel: Experimental design. Brain tissue of WT mice exposed to cotton swab with or without TMT for 30 min was collected at 90 minutes time point, Left lower panel: Quantification of LepR/cFos signal in ARC, DMH and LH, N=4 per group, Unpaired t-test, Two-tailed, Right panel: Representative images of immunohistochemical and in situ hybridization co-staining in female ARC, DMH and LH for LepR (green) and cFos protein (red), yellow cells express LepR/cFos, scale 100µm. All bar graphs are presented as mean values ± SEM. Table S11 contains the detailed results of the statistical analysis. *$p < 0.05$. Source data are provided as a Source Data file.

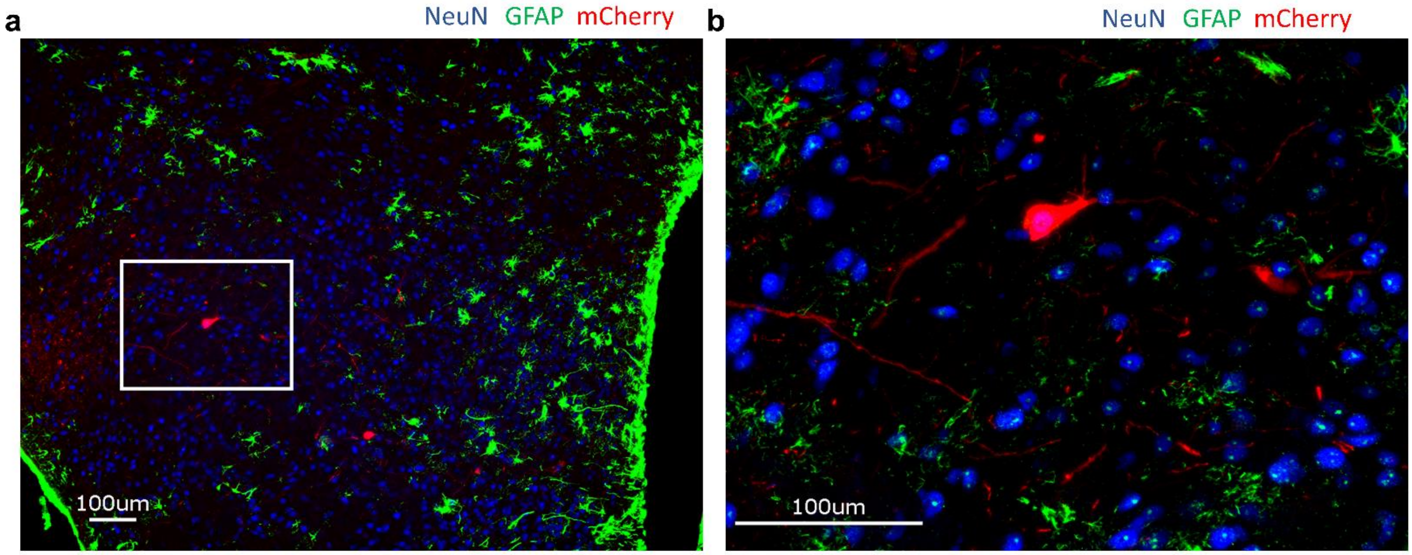

Supplementary figure 6. Anterograde tracing from MOB revels neuronal TdTomato positive cells in hypothalamus. a,b. Immunostaining of NeuN (blue), GFAP (green) and mCherry (red) in mediobasal hypothalamus, Two independent experiments (N=5), scale 100µm.

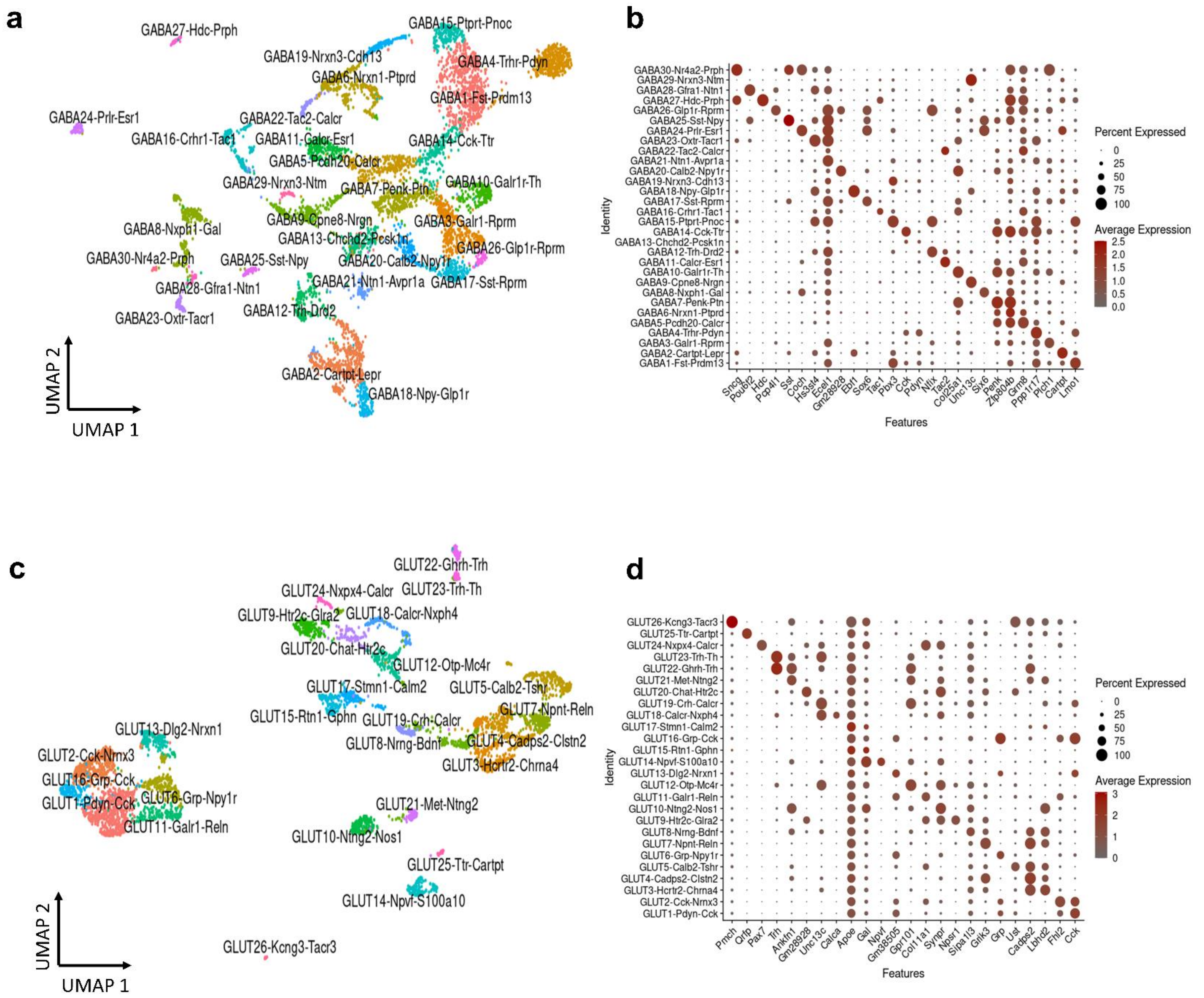

Supplementary figure 7. Single-cell analysis of dorsomedial hypothalamus. a. Uniform Manifold Approximation and Projection (UMAP) plot of inhibitory clusters (5664 cells). **b.** Expression of top genes in each inhibitory cluster. Dot size indicates the percent of nuclei expressing gene and color indicates intensity of expression. **c.** Uniform Manifold Approximation and Projection (UMAP) plot of excitatory clusters (3972 cells). **d.** Expression of top genes in each excitatory cluster. Dot size indicates the percent of nuclei expressing the gene and color indicates intensity of expression. Source data are provided as a Source Data file.

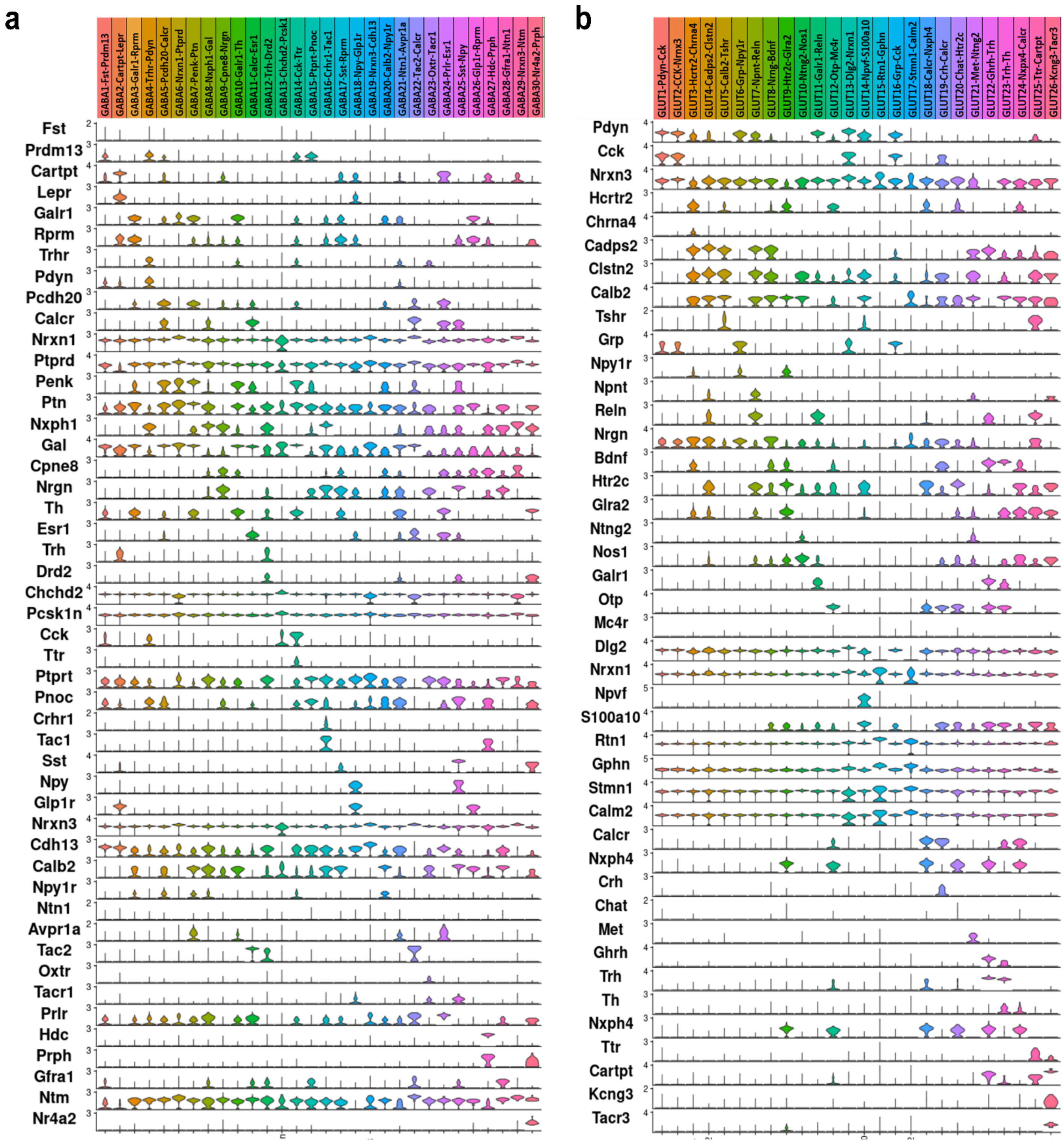

Supplementary figure 8. Representation of cluster nomenclature genes across inhibitory and excitatory clusters. a. Violin plot representing the expression levels of genes used for cluster nomenclature in inhibitory clusters (5664 cells). **b.** Violin plot representing the expression levels of genes used for cluster nomenclature in excitatory clusters (3972 cells). Source data are provided as a Source Data file.

Supplementary Table S1 (related to Figure 1)

b)	Two-way Anova with Tukey's post hoc comparison, N=12-14/group	F_{Sex} (1, 46)=17.54, P=0.0001<br>$F_{Sex\ x\ Treatment}$ (1, 46)=11,32, P=0.0016
c,d)	Three-way Anova, N=16/group	F_{Time} (5.816, 348.9) = 47.56, P<0.0001<br>F_{Sex} (1, 60) = 47.75, P<0.0001<br>$F_{Time\ x\ Treatment}$ (9, 540) = 3.280, P=0.0007<br>$F_{Sex\ x\ Treatment}$ (1, 60) = 7.280, P=0.0090
c)	Two-way Anova with Sidak's post hoc comparison, N=16/group	F_{Time} (5.095, 152.8) = 23.79, P<0.0001<br>$F_{Time\ x\ Treatment}$ (9, 270) = 2.359 P=0.0141
d)	Two-way Anova with Sidak's post hoc comparison, N=16/group	F_{Time}(5.403, 162.1) = 24.27, P<0.0001<br>$F_{Treatment}$ (1, 30) = 8.073, P=0.0080<br>$F_{Time\ x\ Treatment}$ (9, 270) = 1.929, P=0.0481
e,f)	Three-way Anova, N=16/group	F_{Time} (5.010, 300.6) = 21.40, P<0.0001<br>F_{Sex} (1, 60) = 12.44, P=0.0008<br>$F_{Time\ x\ Treatment}$ (9, 540) = 2.790, P=0.0033<br>$F_{Time\ x\ Sex}$ (9, 540) = 3.166, P=0.0010
e)	Two-way Anova with Sidak's post hoc comparison, N=16/group	F_{Time} (4.120, 123.6) = 14.65, P<0.0001<br>$F_{Time\ x\ Treatment}$ (9, 270) = 2.264, P=0.0186
f)	Two-way Anova with Sidak's post hoc comparison, N=16/group	F_{Time} (5.261, 157.8) = 7.471, P<0.0001<br>$F_{Treatment}$ (1, 30) = 4.836, P=0.0357
I,j)	Three-way Anova, N=11/group	F_{Time} (4.733, 189.3) = 6.226, P<0.0001<br>$F_{Time\ x\ Sex\ x\ Treatment}$ (6, 240) = 2.751, P=0.0132
j)	Two-way Anova with Sidak's post hoc comparison, N=11/group	$F_{Time\ x\ Treatment}$ F (6, 120) = 2.680, P=0.0178
k,l)	Three-way Anova, N=9-10/group	F_{Time} F (1.934, 67.69) = 99.12, P<0.0001<br>F_{Sex} (1, 35) = 4.478, P=0.0415<br>$F_{Treatment}$ (1, 35) = 51.28, P<0.0001<br>$F_{Time\ x\ Treatment}$ (5, 175) = 29.21, P<0.0001<br>$F_{Sex\ x\ Treatment}$ (1, 35) = 5.362, P=0.0266
k)	Two-way Anova with Sidak's post hoc comparison, N=9-10/group	F_{Time} (1.557, 26.46) = 49.13, P<0.0001<br>$F_{Treatment}$ F (1, 17) = 8.911, P=0.0083<br>$F_{Time\ x\ Treatment}$ (5, 85) = 3.826, P=0.0036
l)	Two-way Anova with Sidak's post hoc comparison, N=10/group	F_{Time} (2.088, 37.59) = 51.12, P<0.0001<br>$F_{Treatment}$ (1, 18) = 62.98, P<0.0001<br>$F_{Time\ x\ Treatment}$ (5, 90) = 42.82, P<0.0001
o)	Two-way Anova with Dunnett's post hoc comparison, N=5/group	F_{Sex} (1, 32) = 7.739, P=0.0090<br>$F_{Treatment}$ (3, 32) = 8.972, P=0.0002
q)	Two-way Anova with Dunnett's post hoc comparison, N=5/group	$F_{Treatment}$ (3, 32) = 7.887, P=0.0004

Supplementary Table S2 (related to Figure 2)

c)	Two-way Anova with Tukey's post hoc comparison, N=6-8/group	$F_{Treatment}$ (1, 24) = 37.19 , P<0.0001
d)	Two-way Anova with Tukey's post hoc comparison, N=6-11/group	$F_{Treatment}$ (1, 31) = 5.835 , P=0.0218
e)	Two-way Anova with Tukey's post hoc comparison, N=11/group	$F_{Treatment}$ (1, 40) = 15.22, P=0.0004
g,h)	Three-way Anova, N=13-14/group	F_{Time} (5.058, 252.9) = 8.088, P<0.0001<br>F_{Sex} (1, 50) = 8.295, P=0.0058<br>$F_{Treatment}$ (1, 50) = 7.824, P=0.0073<br>$F_{Time\ x\ Treatment\ x\ Sex}$ (8, 400) = 2.631, P=0.0081
h)	Two-way Anova with Sidak's post hoc comparison, N=13/group	F_{Time} (4.943, 118.6) = 7.171, P<0.0001<br>$F_{Treatment}$ (1, 24) = 8.899, P=0.0065<br>$F_{Time\ x\ Treatment}$ (8, 192) = 3.312, P=0.0014
m,n)	Three-way Anova, N=10-17/group	F_{Time} (2.667, 133.4) = 216.6, P<0.0001<br>F_{Sex} (1, 50) = 16.85, P=0.0001<br>$F_{Time\ x\ Sex}$ (8, 400) = 2.631, P<0.0001
p,q)	Three-way Anova, N=8-12/group	F_{Time} (5, 180) = 2.632, P=0.0253<br>F_{Sex} (1, 36) = 31.38, P<0.0001<br>$F_{Time\ x\ Sex}$ (5, 180) = 3.005, P=0.0125
r)	Two-way Anova with Tukey's post hoc comparison, N=7-8/group	F_{Sex} (1, 26) = 16.47 P=0.0004<br>$F_{Treatment}$ (1, 26) = 9.062 , P=0.0057<br>$F_{Sex\ x\ Treatment}$ (1, 26) = 6.294, P=0.0187

Supplementary Table S3 (related to Figure 3)

b)	Two-way Anova with Tukey's post hoc comparison, N=6-12/group	$F_{Surgery}$ (1, 32) = 92.33, P<0.0001
c)	Two-way Anova with Tukey's post hoc comparison, N=8-14/group	$F_{Surgery}$ (1, 38) = 9.875, P=0.0032
d)	Two-way Anova with Tukey's post hoc comparison, N=7-12/group	$F_{Treatment}$ (1, 32) = 4.305 , P=0.0461<br>$F_{Surgery}$ (1, 32) = 19.10 , P=0.0001
f)	Three-way , N=7-13/group	F_{Time} (3.674, 128.6) = 27.23, P<0.0001<br>$F_{Treatment}$ (1, 35) = 11.35, P=0.0019<br>$F_{Time\ x\ Treatment}$ (8, 280) = 3.338, P=0.0011
g)	Two-way Anova with Tukey's post hoc comparison, N=7-13/group	$F_{Treatment}$ (1, 35) = 23.24, P<0.0001

Supplementary Table S4 (related to Figure 4)

a)	Two-way Anova with Tukey's post hoc comparison, N=6-8/group	$F_{Treatment}$ (1, 24) = 22.53 , P<0.0001<br>F_{Sex} (1, 24) = 4.549 , P=0.0434
b)	Two-way Anova with Tukey's post hoc comparison, N=6-7/group	$F_{Treatment}$ (1, 22) = 16.72, P=0.0005
c)	Two-way Anova with Tukey's post hoc comparison, N=6-8/group	$F_{Treatment}$ (1, 24) = 13.16, P=0.0013
d)	Two-way Anova with Tukey's post hoc comparison, N=6-8/group	$F_{Treatment}$ (1, 24) = 19.58 , P=0.0002<br>$F_{Sex\ x\ Treatment}$ (1, 24) = 4.274, P=0.0496
i)	Unpaired t-test,Two-tailed, N=5/group	P=0.0444

Supplementary Table S5 (related to Figure 5)

b)	Two-way Anova with Tukey's post hoc comparison, N=5/group	$F_{Treatment}$ (1, 16) = 48.60 , P<0.0001
e)	Two-way Anova with Tukey's post hoc comparison, N=5/group	$F_{Treatment}$ (1, 16) = 17.23, P=0.0008
i)	Two-way Anova with Tukey's post hoc comparison, N=5/group	$F_{Treatment}$ (1, 16) = 16.36, P=0.0009
j)	Two-way Anova with Tukey's post hoc comparison, N=5/group	$F_{Treatment}$ (1, 16) = 11.61, P=0.0036

Supplementary Table S6 (related to Figure 6)

b)	Model-based Analysis of Single-cell Transcriptomics (MAST)	Cck, P=1.56E-12

Supplementary Table S7 (related to Figure 8).

d)	Unpaired t-test, N=3-4/group	P= 0.0367(males)<br>P= 0.0398(females)
f)	Three-way Anova, N=3-4/group	F_{Time} (2.556, 28.12) = 120.3, P<0.0001<br>F_{Sex} (1, 11) = 10.44, P=0.0080<br>F_{Virus} (1, 11) = 9.974, P=0.0091<br>$F_{Time\ x\ Sex}$ (5, 55) = 13.39, P<0.0001<br>$F_{Time\ x\ Virus}$ (5, 55) = 4.953, P=0.0008
f)	Two-way Anova with Sidak's post hoc comparison, N=3-4/group	Males<br>F_{Time} (2.375, 14.25) = 53.41, P<0.0001<br>$F_{Time\ x\ Virus}$ (5, 30) = 3.006, P=0.0257<br>Females<br>F_{Time} (2.219, 11.10) = 64.28, P<0.0001<br>$F_{Time\ x\ Virus}$ (5, 25) = 2.839, P=0.0257

j)	Three-way Anova, N=8/group<br><br>Statistical significance in time point 22.5 correspond to values on Fig 8k<br><br>For time point 45 Two-way Anova with Tukey's post hoc comparison, N=8/group	F_{Time} (3.377, 94.56) = 8.981, P<0.0001<br>F_{Virus} (1, 28) = 10.16, P=0.0035<br>$F_{Time\ x\ Odor}$ (9, 252) = 6.173, P<0.0001<br>$F_{Time\ x\ Virus}$ (9, 252) = 2.258, P=0.0191<br>$F_{Odor\ x\ Virus}$ F (1, 28) = 0.4890, P=0.4901<br>$F_{Odor\ x\ Virus\ x\ Time}$ F (9, 252) = 0.5650, P=0.8251<br><br>F_{Odor} (1, 28) = 23.45, P<0.0001<br>F_{Virus}(1,28)= 15.01, P=0.0006
k)	Two-way Anova with Tukey's post hoc comparison, N=8/group VO2 (t=22.5min)	F_{Virus} (1, 28) = 15.01, P=0.006<br>F_{Odor} (1, 28) = 23.45, P<0.0001
l)	Three-way Anova, N=8/group<br><br>Statistical significance in time point 22.5 correspond to values on Fig 8m	F_{Time} (5.698, 159.5) = 8.475, P<0.0001<br>$F_{Time\ x\ Odor}$ (9, 252) = 3.576, P=0.0003
m)	Two-way Anova with Tukey's post hoc comparison, N=8/group Activity (t=22.5min)	F_{Odor} (1, 28) = 13.01, P=0.0012
n)	Three-way Anova, N=6-8/group	F_{Time} (2.298, 55.14) = 105.4, P<0.0001<br>F_{Odor} (1, 24) = 13.91, P=0.0010<br>F_{Virus} (1, 24) = 5.302, P=0.0303<br>$F_{Time\ x\ Odor}$ (5, 120) = 6.769, P<0.0001<br>$F_{Time\ x\ Virus}$ (5, 120) = 3.171, P=0.0101
o)	Two-way Anova with Tukey's post hoc comparison, N=6-8/group	F_{Virus} (1, 24) = 4.484, P=0.0448<br>F_{Odor} (1, 24) = 10.83, P=0.0031

Supplementary Table S8 (related to Supplementary Figure 1)

b)	Two-way Anova with Sidak's post hoc comparison , N=4-10/group	$F_{Treatment}$ (1, 24) = 21.98,<br>P<0.0001
c)	Two-way Anova with Sidak's post hoc comparison , N=4-5/group	$F_{Treatment}$ (3, 31) = 60.48, P<0.0001<br>$F_{Treatment\ x\ Sex}$ (3, 31) = 4.335, P=0.0113
d)	Two-way Anova with Sidak's post hoc comparison , N=4-5/group	$F_{Treatment}$ (3, 32) = 23.79, P<0.0001

Supplementary Table S9 (related to Supplementary Figure 2)

c)	ANCOVA	Response: EE<br>Sum Sq Df F value Pr(>F)<br>(Intercept) 0.000925 1 0.7453 0.3923<br>group 0.002588 3 0.6948 0.5598<br>BW 0.001602 1 1.2901 0.2617<br>group:BW 0.001620 3 0.4350 0.7289<br>Residuals 0.059596 48

d)	Two-way Anova with Sidak's post hoc comparison , N=13 group, Results for 3 hours post CNO injection	F_{Time} (4.943, 118.6) = 7.171, P<0.0001<br>$F_{Treatment}$ (1, 24) = 8.899, P=0.0065<br>$F_{Time\ x\ Treatment}$ (8, 192) = 3.312, P=0.0014
h)	Two-way Anova with Sidak's post hoc comparison , N=12-14/group	Day 1<br>$F_{Treatment}$ (1, 50) = 8.295, P=0.0058<br>F_{Sex} (1, 50) = 7.824, P=0.0073<br>Day 2<br>$F_{Treatment}$ (1, 50) = 11.91, P=0.0011<br>F_{Sex} (1, 50) = 37.65, P<0.0001<br>Day 1<br>$F_{Treatment}$ (1, 43) = 6.619, P=0.0136<br>F_{Sex} (1, 43) = 37.09, P<0.0001
i)	Two-way Anova with Tukey's post hoc comparison , N=8-10/group	F_{Sex} (1, 32) = 26.29, P<0.0001

Supplementary Table S10 (related to Supplementary Figure 3)

a)	Two-way Anova with Tukey's post hoc comparison , N=4-10/group	$F_{Treatment}$ (1, 24) = 26.31, P<0.0001

Supplementary Table S11 (related to Supplementary Figure 5)

b)	Unpaired t test, Two-tailed, N=4/group	P=0.0303
