# Supplementary material for: A sex-specific thermogenic neurocircuit induced by predator smell recruiting cholecystokinin neurons in the dorsomedial hypothalamus
Source: Nat Commun. 2023 Aug 15;14:4937. doi: 10.1038/s41467-023-40484-7 (PMC10427624; doi:10.1038/s41467-023-40484-7)
Supplement: Supplementary file 3 — Description of Additional Supplementary Files [file 41467_2023_40484_MOESM3_ESM.pdf]

Description of Additional Supplementary Files

Supplementary Movie 1. Light sheet imaging of the cleared brain upon OBTbx21chemogenetic activation. iDISCO tissue clearing was applied to a Cre+-hM3Dq+ female brain and immunostaining was performed for cFos (red). Green channel was used as autofluorescence.
