# Supplementary material for: A sex-specific thermogenic neurocircuit induced by predator smell recruiting cholecystokinin neurons in the dorsomedial hypothalamus
Source: Nat Commun. 2023 Aug 15;14:4937. doi: 10.1038/s41467-023-40484-7 (PMC10427624; doi:10.1038/s41467-023-40484-7)
Supplement: Supplementary file 5 — Reporting Summary [file 41467_2023_40484_MOESM5_ESM.pdf]

nature portfolio

Corresponding author(s): Celine Riera

Last updated by author(s): Mar 27, 2023

Reporting Summary

Nature Portfolio wishes to improve the reproducibility of the work that we publish. This form provides structure for consistency and transparency in reporting. For further information on Nature Portfolio policies, see our Editorial Policies and the Editorial Policy Checklist.

Statistics

For all statistical analyses, confirm that the following items are present in the figure legend, table legend, main text, or Methods section.

n/a	Confirmed	
[ ]	[x]	The exact sample size (*n*) for each experimental group/condition, given as a discrete number and unit of measurement
[ ]	[x]	A statement on whether measurements were taken from distinct samples or whether the same sample was measured repeatedly
[ ]	[x]	The statistical test(s) used AND whether they are one- or two-sided<br>*Only common tests should be described solely by name; describe more complex techniques in the Methods section.*
[x]	[ ]	A description of all covariates tested
[ ]	[x]	A description of any assumptions or corrections, such as tests of normality and adjustment for multiple comparisons
[ ]	[x]	A full description of the statistical parameters including central tendency (e.g. means) or other basic estimates (e.g. regression coefficient) AND variation (e.g. standard deviation) or associated estimates of uncertainty (e.g. confidence intervals)
[ ]	[x]	For null hypothesis testing, the test statistic (e.g. *F*, *t*, *r*) with confidence intervals, effect sizes, degrees of freedom and *P* value noted<br>*Give P values as exact values whenever suitable.*
[x]	[ ]	For Bayesian analysis, information on the choice of priors and Markov chain Monte Carlo settings
[x]	[ ]	For hierarchical and complex designs, identification of the appropriate level for tests and full reporting of outcomes
[x]	[ ]	Estimates of effect sizes (e.g. Cohen's *d*, Pearson's *r*), indicating how they were calculated

Our web collection on statistics for biologists contains articles on many of the points above.

Software and code

Policy information about availability of computer code

Data collection	Indirect calorimetry data were collected with Phenomaster v.6.6.1.<br>Image collection was performed with Keyance BZ-X710 microscope and processed with BZX Analyzer v.1.3.1.1.<br>Open field test data were collected with Photobeam Activity System (San Diego Instruments).<br>Thermographic images were collected with IR camera FLIR E54 (FLIR System).<br>Mitochondrial activity data were collected by Agilent Seahorse XF24 Analyzer
Data analysis	GraphPad Prism v.8.4.3 for Windows (GraphPad Softwear, LLC) was used for statistical analysis and plotting.<br>Open Field test data were analyzed with Photobeam Activity System Reporter and PAS PathView software v.1.0.2.7.<br>Thermographic images were analyzed with FLIR Tools Thermal Analysis and Reporting Software v.5.13.18031.2002<br>Agilent Seahorse XF24 Analyzer v2.6 analyzed mitochondrial activity data.<br>ROI confirmation was performed with QuickNII v.3-2017.<br>Counting of positive cells within ROI was performed with ImageJ v.1.53k<br>Analysis of cells co-expressing cFos and some of the tested genes was performed by QuPath v.0.3.2 and v.0.4.0<br>3D reconstruction of the whole mouse brain was performed with Imaris v.9.6.1<br>Sequencing data aligning and generating matices was performed with 10x Genomics Cell Ranger v.6.0.0 count pipeline<br>scRNA-seq data were analyzed in R v.4.1.2 using Seurat v.4.1.0.9007<br>Expression count for Bulk RNA was performed using STAR v.2.6.1<br>Acnova was performed in R v.4.1.2 with modified code available at https://www.datanovia.com/en/lessons/ancova-in-r/

For manuscripts utilizing custom algorithms or software that are central to the research but not yet described in published literature, software must be made available to editors and reviewers. We strongly encourage code deposition in a community repository (e.g. GitHub). See the Nature Portfolio guidelines for submitting code & software for further information.

Data

Policy information about availability of data

All manuscripts must include a data availability statement. This statement should provide the following information, where applicable:

- Accession codes, unique identifiers, or web links for publicly available datasets
- A description of any restrictions on data availability
- For clinical datasets or third party data, please ensure that the statement adheres to our policy

All data generated and analyzed during this study are included in this article and its supplementary information files. All sc-RNA seq data of mouse dorsomedial hypothalamus that are used in this study have been deposited in the the National Center for Biotechnology Information Gene Expression Omnibus (GEO) and are accessible through the GEO Series accession number: GSE232230. Mouse reference transcriptome v.2 used in scRNA-seq analysis is available for download at www.thepoollab.org/resources and custom mouse reference genome GRCm38.p5 used for bulk RNA-seq can be found under RefSeq assembly accession: GCF_000001635.25. Source data are provided with this paper.

Human research participants

Policy information about studies involving human research participants and Sex and Gender in Research.

Reporting on sex and gender	N/A
Population characteristics	N/A
Recruitment	N/A
Ethics oversight	N/A

Note that full information on the approval of the study protocol must also be provided in the manuscript.

Field-specific reporting

Please select the one below that is the best fit for your research. If you are not sure, read the appropriate sections before making your selection.

☒ Life sciences ☐ Behavioural & social sciences ☐ Ecological, evolutionary & environmental sciences

For a reference copy of the document with all sections, see nature.com/documents/nr-reporting-summary-flat.pdf

Life sciences study design

All studies must disclose on these points even when the disclosure is negative.

Sample size	We did not employ any formal statistical method to determine the sample size. Instead, we based our sample size on the prior research that employed similar methodologies (Makwana K, Chodavarapu H, Morones N, et al. Sensory neurons expressing calcitonin gene-related peptide α regulate adaptive thermogenesis and diet-induced obesity. Molecular Metabolism. 2021;45:101161). For the majority of our experiments, we examined at least five independent biological replicates per condition. In certain experiments, we analyzed a smaller number of replicates (n=3-4), but found that this sample size adequately accounted for biological variability, particularly when the data was highly reproducible.
Data exclusions	Animals in which we failed to confirmed viral expression on both sites were excluded from experiments that required bilateral viral injection. For single cell RNA-seq experiments common thresholding to remove doublets and unhealthy cells was implemented. The food intake data collected by Phenomaster for the cages where food shredding was observed were excluded from the analysis.
Replication	In vivo experiments were performed at least two independent times to confirm and successfully validate reproducibility. All replication were included in data.
Randomization	All animals were randomly assigned to different groups.
Blinding	The behavioral data were collected in a double-blind fashion to minimize potential sources of bias. The majority of the data were subjected to automated analysis using software. In instances where manual analysis was necessary, the investigators were blinded to the treatment conditions to prevent subjective bias.

Reporting for specific materials, systems and methods

We require information from authors about some types of materials, experimental systems and methods used in many studies. Here, indicate whether each material, system or method listed is relevant to your study. If you are not sure if a list item applies to your research, read the appropriate section before selecting a response.

Materials & experimental systems

n/a	Involved in the study
☐	☒ Antibodies
☒	☐ Eukaryotic cell lines
☒	☐ Palaeontology and archaeology
☐	☒ Animals and other organisms
☒	☐ Clinical data
☒	☐ Dual use research of concern

Methods

n/a	Involved in the study
☒	☐ ChIP-seq
☒	☐ Flow cytometry
☒	☐ MRI-based neuroimaging

Antibodies

Antibodies used	Primary antibodies used in this study were:anti-NeuN (1:500, PA5-78499, Thermo Fisher Scientific), anti-mCherry (1:500, PA5-34974, Thermo Fisher Scientific), anti-cFos (1:1000 and 1:5000, ab190289, Abcam), anti-GFAP (1:1000, 173004, Synaptic Systems). The secondary ab used: : donkey anti-rabbit Alexa Fluor® 488(1:500, A21206, Thermo Fisher Scientific), donkey anti-rabbit Alexa Fluor® 594 (1:500, A21207, Thermo Fisher Scientific), goat anti-guinea pig Alexa Fluor® 488 (1:500, A11073, Thermo Fisher Scientific),goat anti-rabbit Alexa Fluor® 405 (1:500, A48254, Thermo Fisher Scientific) and donkey anti-rabbit Alexa Fluor® 647 ((1:5000, ab 150075, Abcam)
Validation	anti-NeuN, PA5-78499, Thermo Fisher Scientific, This Antibody was verified by Relative expression to ensure that the antibody binds to the antigen stated, 5 citations<br>anti-mCherry, PA5-34974, Thermo Fisher Scientific, 40 citations<br>anti-cFos, ab190289, Abcam, 140 citations<br>anti-GFAP, 173004, Synaptic Systems, 72 citations

Animals and other research organisms

Policy information about studies involving animals; ARRIVE guidelines recommended for reporting animal research, and Sex and Gender in Research

Laboratory animals	In this study, we used Ai14 (Jax strain 007914), Tbx21-Cre (Jax strain 024507), R26-LSL-hM3Dq-DREADD (Jax strain 026220), Cck-IRES-Cre (Jax strain 012706), and C57BL/6J (Jax strain 000664). 8-12 weeks old mice were used.Mice were maintained on a 14 h: 10 h light:dark cycle, fed normal chow (PicoLab Rodent 20 5053*, LabDiet). All experiments are done at 22°C, humidity 30-70%.
Wild animals	No wild animals were used in the study.
Reporting on sex	In all experiments, we used both sexes, except the experiment with the silencing of Cck-expressing neurons in DMH, where only females were used. The variable of sex was incorporated and documented in the statistical analyses performed (2-way and 3-way ANOVA).
Field-collected samples	No field collected samples were used in the study.
Ethics oversight	All procedures were approved by the Animal Care and Use Committee of Cedars Sinai Medical Center.

Note that full information on the approval of the study protocol must also be provided in the manuscript.
